# Transgenerational epigenetic effects from male exposure to endocrine-disrupting compounds: a systematic review on research in mammals

**DOI:** 10.1186/s13148-020-00845-1

**Published:** 2020-05-12

**Authors:** Olivia Van Cauwenbergh, Alessandra Di Serafino, Jan Tytgat, Adelheid Soubry

**Affiliations:** 1 Epidemiology Research Center, Department of Public Health and Primary Care, Faculty of Medicine, KU Leuven - University of Leuven, Leuven, Belgium; 2 Department of Psychological, Health and Territorial Sciences, School of Medicine and Health Sciences, University “G.d’Annunzio” of Chieti-Pescara, Chieti, Italy; 3 Toxicology and Pharmacology, Department of Pharmaceutical and Pharmacological Sciences, KU Leuven - University of Leuven, Leuven, Belgium

**Keywords:** Epigenetic toxicity, Endocrine disruptors, Father, Sperm, Inheritance

## Abstract

Assessing long-term health effects from a potentially harmful environment is challenging. Endocrine-disrupting compounds (EDCs) have become omnipresent in our environment. Individuals may or may not experience clinical health issues from being exposed to the increasing environmental pollution in daily life, but an issue of high concern is that also the non-exposed progeny may encounter consequences of these ancestral exposures. Progress in understanding epigenetic mechanisms opens new perspectives to estimate the risk of man-made EDCs. However, the field of *epigenetic toxicology* is new and its application in public health or in the understanding of disease etiology is almost non-existent, especially if it concerns future generations. In this review, we investigate the literature on transgenerational inheritance of diseases, published in the past 10 years. We question whether persistent epigenetic changes occur in the male germ line after exposure to synthesized EDCs. Our systematic search led to an inclusion of 43 articles, exploring the effects of commonly used synthetic EDCs, such as plasticizers (phthalates and bisphenol A), pesticides (dichlorodiphenyltrichloroethane, atrazine, vinclozin, methoxychlor), dioxins, and polycyclic aromatic hydrocarbons (PAHs, such as benzo(a)pyrene). Most studies found transgenerational epigenetic effects, often linked to puberty- or adult-onset diseases, such as testicular or prostate abnormalities, metabolic disorders, behavioral anomalies, and tumor development. The affected epigenetic mechanisms included changes in DNA methylation patterns, transcriptome, and expression of DNA methyltransferases. Studies involved experiments in animal models and none were based on human data. In the future, human studies are needed to confirm animal findings. If not transgenerational, at least intergenerational human studies and studies on EDC-induced epigenetic effects on germ cells could help to understand early processes of inheritance. Next, toxicity tests of new chemicals need a more comprehensive approach before they are introduced on the market. We further point to the relevance of epigenetic toxicity tests in regard to public health of the current population but also of future generations. Finally, this review sheds a light on how the interplay of genetics and epigenetics may explain the current knowledge gap on transgenerational inheritance.

## Background

### A long-lasting story about widespread applications and safety of man-made EDCs

An extensive warning about adverse biological effects from environmental exposures to endocrine-disrupting compounds (EDCs) came as early as 1962, through a book entitled “Silent Spring.” In this work, Rachel Carlson argued that the use of pesticides might cause grave danger on human and animal health, especially through bioaccumulation in the food chain [1]. Her warnings regarding harmful contributions of humans to the environment and the consequences hereof on health of living species of all kinds have been evidenced in numerous studies and still resonate high today. Nearly 50 years later, the urgency of her warnings still persists due to the use of a variety of EDCs in agricultural and industrial applications, the paramount increase of widespread EDCs, and their accumulation in nature. For instance, pesticides have been designed to be toxic to pests’ nervous or reproductive system, but they also possess the potential of interfering with physiological or hormonal processes in human reproductive health, resulting in reproductive pathologies, infertility, metabolic and neurologic disorders; studied and discussed by many researchers [2–5]. Today, more than 100,000 substances are commercially available in Europe alone. This number is growing rapidly, since new chemicals are being introduced every day [6]. Of particular concern is the fact that a large number of man-made EDCs leak into our in- and outdoor environment, water reservoirs, seas, and soils [7]. Traces often remain in our environment for many years, even after the original chemical has been banned. Consequently, their levels, decay, fate, and especially their toxicity for human beings remain of current concern. While it is well known that EDCs play a role in the etiology of several chronic diseases, the fact that people are exposed to mixtures of chemicals throughout their lives—through different routes—increases their risk for adult-onset diseases even more. Importantly, EDCs in consumer products do not need to pass the same safety tests as those applied for drugs and foods. However, some pollutants have been traced for absorbance into the human body [8–10]. A comprehensive framework of approaches to define and characterize the risks of EDCs is indispensable. The EU defines EDCs as “an exogenous substance that causes adverse health effects in an intact organism, or its progeny, secondary to changes in endocrine function” [11]. Endocrine toxicity is detected through applying the OECD (Organization for Economic Co-operation and Development) guidelines, including rodent two-generation reproduction tests, extended one-generation reproductive toxicity studies, rodent chronic toxicity and oncogenicity tests, and 28-day toxicity analyses [12]. However, standard toxicity tests do not involve long-term effects, such as inheritance of diseases across generations. Especially if no adverse health effects are noticeable in the exposed organism(s), there is generally no reason for concern.

New findings based on the latest technological developments in the field of environmental epigenetics propose that early exposures may be transmitted transgenerationally through molecular changes in the germ line. Importantly, these non-genetic effects may remain subclinical at first. In order to be prudent regarding potential health consequences on the longer term and taking into account new developments in the field of environmental epigenetics, we believe that OECD guidelines need further elaboration.

### Epigenetic inheritance of diseases caused by exposure to EDCs: involvement of the male germ line

Increasing evidence shows that environmental factors—of all kinds—may induce functional changes of the genome. In this way, related disorders may be inherited from father to child. This process is driven by epigenetic components of the cell. Changes in DNA methylation, histone modification, and non-coding RNAs are viable mechanistic candidates for a non-genetic transfer of paternal environmental information, from maturing germ cells to zygote. We earlier suggested the existence of epigenetic windows of susceptibility to environmental insults during sperm development [13]. Male gametes are potentially at higher risk for epigenetic damage during their epigenetic reprogramming periods, and environmental factors can alter the fidelity of this process. Consequently, these “environmental messages” are likely transmitted to the next generation(s). While most evidence is based on animal models, some studies show that sperm from a general human population is susceptible to environmental traits, including an unhealthy lifestyle or obesity [14] and exposure to pollutants, such as organophosphates [15]. In 2013, we provided the first indication that epigenetic signatures can be transferred in human, from father to child [16, 17]. Others have published similar findings in human study populations [18]. Some trans- or intergenerational effects from early exposures have been found in humans through longitudinal study designs. For instance, a sex-specific increased risk for mortality was found in grandchildren of grandmothers who had been exposed to an excess of food supply during prepuberty; and, a link between obesity in adolescent boys was found if the father started smoking at an early age [19, 20]. Interestingly, paternal exposure to phthalates has been associated with poor blastocysts quality in couples attending the fertility clinic [21], and a link with sperm DNA methylation aberrancies has been suggested [22]. Although no epigenetic mechanisms have been confirmed yet as a potential underlying mechanism, others and us have discussed this possibility in earlier publications [13, 23–25]. This has led to the introduction of new theory: the Paternal Origins of Health and Disease (POHaD) paradigm [26, 27].

EDCs belong to one of the many traits from our environment that may compromise health of current or next generations through epigenetic reprogramming. While this idea is not new [28], we believe that this research topic is underexplored and needs more attention. Exposure to EDCs increases risk for metabolic diseases, obesity, and many other disorders [5, 29, 30]. Although the plasticity of the epigenome provides a plausible explanation for inheritance of these phenotypes [31], the exact mechanisms are still not completely known, and substantial data from human studies are missing. Furthermore, epigenotoxicity tests—as suggested by Bernal et al. in 2010 [31]—have not been implemented yet.

While we believe that epigenetic inheritance through the male germ line is a plausible hypothesis to explain how signals from early exposures, such as to EDCs, can be transferred through generations [27], yet, no systematic reviews have been performed to estimate the importance of epigenetic inheritance in living species. Literature distinguishes at least two kinds of inheritance. First, *intergenerational inheritance*, which originates from direct exposures, where a germ cell receives external signals and translates these into epigenetic changes. If these changes are able to persist after fertilization and early development, phenotypic or metabolic variation occurs and risk for disease may be different in the offspring, compared to its (grand)parents. For instance, when a pregnant mother (F0) is exposed to an adverse environmental factor, the offspring (F1) as well as the grandchildren (F2) may be affected as a consequence of the in-utero exposure in the developing embryo (F1) or the developing germ cell (later F2). Alternatively, direct exposure in the life of the father (F0) may affect his offspring (F1) through epigenetic changes in his sperm. Second, *transgenerational inheritance* is a phenomenon where the effects are manifested in the unexposed generation [32]. If exposed in-utero, the F3 generation would be the first generation that acquires a transgenerational phenotype. If the exposure occurs in life (preconceptionally), effects in the F2 generation are also called transgenerational. Interestingly, although their mechanism has not yet been fully described, epigenetic effects that are transgenerational may be at the origin of persistent and evolutionary important changes.

The aim of this review is to increase our understanding of the mechanism of action by which EDCs affect the next generation through the male epigenome, in a trans- or intergenerational way. Results of this systematic review will provide evidence-based knowledge on EDCs and their role in disease inheritance, providing an opportunity to modulate risk of future offspring by intervention and/or prevention strategies in future fathers. Next, if evidence is convincing enough, epigenetic inheritance should be taken into consideration when toxicity tests are implemented.

## Methods

### Search strategy and study selection

**Fig. 1:**
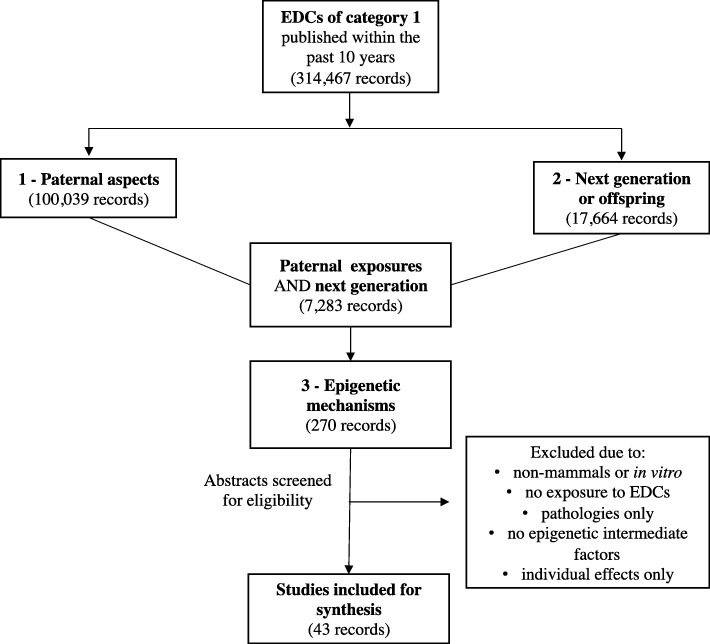
Study identification and selection process. Our search resulted in 314,467 records on EDCs. From these, 100,039 articles were on concept 1 (paternal aspects) and 17,664 were on concept 2 (next generation or offspring). The number of articles on associations between concept 1 and concept 2 resulted in 7283 articles. Inclusion of concept 3 (epigenetics) resulted 270 articles. Finally, 43 articles met our inclusion criteria and were considered in the present study

We conducted a systematic review of the literature on potential effects of exposures to endocrine disrupting chemicals (EDCs). Substances were compiled from the official European Union list of potential EDCs and were also accessible through the website of the Ministry of Environment and Food of Denmark [33, 34]. Supplementary Table 1 shows EDCs of category 1, meaning that their endocrine-disrupting effects were documented in at least one study of a living organism. These substances are given the highest priority for further studies by the EU. Substances of category 2 and 3 (not shown) are chemicals with less or no indications of endocrine-disrupting properties. We performed our search in February 2019, using the advanced search builder of the PubMed database. We filtered hits by selecting articles published over the last 10 years (from 2008/01/01 to 2019/02/22), written in English, and excluding reviews. Our search included comprehensive Medical Subject Heading (MeSH) terms on three different concepts: 1/ *paternal aspects*, 2/ *next generation or offspring outcome*s, and 3/ *epigenetic inheritance*. A free search through title and abstract was added to obtain the latest articles. Moreover, because of the novelty of our topic, some MeSH-terms may not yet be assigned to our subject. Hence, we added the following keywords for concept 1: paternal, male, man, men, infertility, sterility, subfertility, spermatoz*, sperm*, semen, and adult germ line stem cells. Keywords for concept 2 included progeny, offspring*, generation*, newborn*, child*, neonate*, infant, baby, babies, male pup, litter, embryo, fetus, perinatal exposure, prenatal exposure, prenatal injuries, father-child relations. Keywords for concept 3 included intergenerational, transgenerational, epigen*, epimutation, genomic instability, DNA methylation, methylated, CpG*, microRNA*, histone modification*. Two independent investigators extracted articles and determined eligibility. A third investigator resolved potential discrepancies. Our study selection process is depicted in Fig. 1, starting with a screening for EDCs we selected based on the EU list of category 1 EDCs (Table 1). Next, we searched for studies on exposure to (future) fathers and male gametes (concept 1). Additionally, we searched for articles on next generation (concept 2). Finally, research articles exploring also epigenetic mechanisms were selected for further assessment (concept 3). In our final screening, we excluded studies that were not related to mammals (e.g., zebrafish) or that did not study potential effects from male-related EDC exposures (e.g., if similar substances were used in methods or protocols). Next, studies that solely focussed on direct effects on male germ cells of the exposed individual were not included.

**Table 1:** List of 43 selected articles by EDC exposures, including study design and main findings

EDC	Administration of the exposure; animal model	Samples tested	Main findings	Reference
Atrazine	Orally, during pregnancy from E6.5 till E15.5 (100 mg/kg/day); CD1 mice	F1: testes (E15.5 and E18.5) F3: testes, liver, hypothalamus	F3 testes: decreased sperm number, meiotic defects F1 and F3 testes: histone modifications (H3K4me3) F3 somatic cells: RNA expression that corresponds to histone modification in F1 sperm	Hao et al. 2016 [35]
	Daily ip injections, from E8 till E14 (25 mg/kg); Hsd:SD1 rats	F1, F2, F3: epididymal sperm F1, F2, F3: testis, epididymis, prostate, ovary, kidney (12 months)	F1: lean, but no diseases F2: lean females, mammary tumors, testis diseases, early-onset puberty in males F3: lean and similar disease risk as F2, motorhyperactivity	McBirney et al. 2017 [36]
Benzo[a]Pyrene (B[a]P)	Single ip injection prior to IVF treatment (150 mg/kg); B6D2F1 mice	Embryos (2-cell, 8-cell, blastocyst)	Differential miRNA expression patterns in embryos by cell-stage and (B[a]P) exposure	Brevik et al. 2012 [37]
	Single ip injection, 4 days prior to IVF treatment (150 mg/kg); B6D2F1 mice	Embryos (1-cell, 2-cell, 4-cell, 8-cell, blastocyst)	Several genes were differentially expressed in response to B[a]P exposure Functional analysis showed that paternal B[a]P exposure triggers biological processes, such as DNA transcription, DNA damage response, cell cycle regulation, chromatin modification, oxidation-reduction processes, apoptosis, and embryo development	Brevik et al. 2012 [38]
	During 6 weeks (3 times per week) oral doses of B[a]P (13 mg/kg); C57BL/6 male mice crossed with Balb/c wildtype female mice	F1: liver (PN21)	Paternal exposure to B[a]P can regulate the male offspring's mitochondrial stress levels. Proteins involved in mitochondrial function were downregulated. This was paralleled by a reduction in mDNA copy number and reduced activity of citrate synthase and b-hydroxyacyl-CoA dehydrogenase. Both 8-oxo-dG and MDA-dG adduct levels were reduced. miRNA-122, miRNA-129-2-5p, and miRNA-1941 were upregulated in a gender-specific manner	Godschalk et al. 2018 [39]
Bisphenol A (BPA)	Orally, during pregnancy and lactation, from GD0 to PND21 (40 μg/kg/day); SD rats	F1: sperm F2: blood, liver	DNA methylation changes in F1 sperm and in F2 liver, although not similar Hypermethylation of Gck promoter and altered gene expression in liver of F2 rats	Li et al. 2014 [40]
	Orally, during pregnancy and lactation, from GD0 to PND21 (40 μg/kg/day); SD rats	F1: sperm F2: blood, pancreatic islets	Pancreatic β-cell dysfunction and glucose intolerance Increased DNA methylation at *Igf2* DMR2 in F1 sperm Decreased Igf2 expression in F1 sperm DNA hypermethylation of *Igf2* in pancreatic islets in the F2 generation	Mao et al. 2015 [41]
	Diet, 2 weeks prior to mating until weaning, 2 doses: 10 μg/kg/day and 10mg/kg/day); C57BL/6J mice	F1: pancreatic islets (16–21 weeks) F2: pancreatic islets (adult)	F1 and F2 males: impaired insulin secretion and increased levels of pro-inflammatory cytokines Dose- and sex-specific effects in gene expression levels related to inflammation and mitochondrial function, in F1 and F2 Altered DNA methylation at *Igf2* DMR1 and increased *Igf2* expression in F1 and F2	Bansal et al. 2017 [42]
	Diet of females supplemented with BPA (5 mg/kg), 10 days before mating until end of gestation; C57BL/6 mice (note: males were also exposed to the same diet during 2 weeks of mating) Second model: Oral administration of 3 doses (0.5, 20, or 50 μg/kg/day) from E11 till birth; FVB mice	F3 pups (PN4): brain	50 differentially regulated genes were identified in the F3 brain of exposed lineages. A selected imprinted gene, *Meg3*, was upregulated Similar results were found in both models (C57BL/6J mice and FVB mice)	Drobna et al. 2018 [43]
Dichlorodiphenyltrichloroethane (DDT)	Daily ip injections, from E8 till E14 (25 or 50 mg/kg); Hsd:SD1 rats	F1-F4 (10–12 months): multiple organs (testis, epididymis, seminal vesicle, prostate, kidney, ovary and uterus)	Several disorders emerged in the F3 generation, including obesity, testis disease, polycystic ovarian disease, immune abnormalities and kidney disease DNA methylation at numerous DMRs was affected in F3 sperm	Skinner et al. 2013 [44]
	Daily ip injections, from E8 till E14 (25 mg/kg); Hsd:SD rats	F1, F2, F3: epididymal sperm (PN120)	F1, F2: altered DNA methylation and ncRNA F3: novel histone retention sites, compared to F1 and F2. Cellular apoptosis in testes	Skinner et al. 2018 [45]
	Daily ip injections, from E8 till E14 (25 mg/kg/day); Hsd:SD rats	F3: epididymal sperm (PN120)	F3: induced H3 differential histone retention sites (DHRs); while a core histone retention sites were not altered.	Ben Maamar et al. 2018 [46]
	Daily ip injections, from E8 till E14 (25 mg/kg); Hsd:SD rats	F3: prospermatogonia (E16), spermatogonia (P10); and adult pachytene spermatocytes, round spermatids, caput epididymal spermatozoa, and caudal sperm (12 months)	F3: DNA methylation alterations of DMRs were identified at each stage, but the majority were found in (pro)spermatogonia. A link with metabolic and cancer related pathways was shown in all stages	Ben Maamar et al. 2019 [47]
Dioxins and dioxin-like compounds				
2,3,7,8-Tetrachlorodibenzo-p-dioxin (TCDD)	Daily ip injections, from E8 till E14 (100 ng/kg); Hsd:SD rats	F1: testis, prostate, ovary, uterus, kidney F3: testis, prostate, ovary, uterus, kidney, and epididymal sperm (PN120)	F1: increased prostate disease, ovarian primordial follicle loss, polycystic ovary disease F3: increased kidney disease in males, ovarian pubertal abnormalities, primordial follicle loss, polycystic ovary disease F3 sperm: altered DNA methylation at 50 DMRs	Manikkam et al., 2012 [48]
	Orally, during pregnancy from E8 till E14 (200 or 800 ng/kg); SD rats	F1, F3: hepatic tissue (PN90)	F1, F3: decreased *Igf2* expression, hepatic damage, increased activity of hepatic enzymes, hypermethylated ICR of *Igf2*, hypomethylated DMR2 near *H19*, changes in expression of DNMTs	Ma et al. 2015 [49]
	Orally, single dose during pregnancy on E15.5 (10 μg/kg); C57BL/6 mice	F1, F3: male-derived placentae F1, F3: epididymal sperm	F1, F3: >2000 differentially methylated regions in placenta, including *Igf2* and *Pgr*; methylation and expression of the latter was also altered in F1/F3 sperm and F3 placenta	Ding et al. 2018 [50]
P,p′-DDE	Orally, from E8 till E15 (100 mg/kg/day); SD rats	F1, F3, F3: motile sperm (swim-up), testes (E18 and PN120), pancreas (8 weeks)	F1, F2, F3: modifications at DMRs of imprinted genes: *IGF2*/*H19* and *Gtl2* hypomethylation. These genes were upregulated in sperm and testis. Impaired glucose tolerance, abnormal insulin secretion and β-cell dysfunction. Pancreatic impairment and decreased sperm characteristics in offspring (F3) of exposed grandfathers (F1 *in utero*). DNMT1 and 3a were decreased in embryonic testis of F1 and F2 (but not in F3)	Song et al. 2014 [51] Song et al. 2017 [52] Song et al. 2018 [53]
Methoxychlor (MXC)	Daily ip injections (10 mg/kg) in adult males (8 weeks old), during 8 days Daily ip injections, from E8 till E10 (10 mg/kg); FVB/N mice	F1, F2, F3: tail, liver, skeletal muscle, epididymal sperm (2 months)	F1: decreased mean sperm concentrations, altered DNA methylation patterns at several imprinted genes in sperm F2-F3: transference of defects through the male germ line, but methylation defects were limited to a few genes	Stouder et al. 2011 [54]
	Daily ip injections, from E8 till E14 (200 mg/kg); Hsd:SD1 rats	F1, F3, F4: kidney, ovary, uterus, testis, prostate, epididymal sperm (10-12 months)	F1, F3: increased incidence of kidney disease, ovary disease, obesity and multiple diseases F3: sperm "epimutations" F4: increased disease incidence through the female germ line	Manikkam et al. 2014 [55]
Phthalates	Daily ip injections, from E7 till E19 (750 mg/kg); SD rats	F1, F2, F3, F4: testis, epididymal sperm (PN80)	F1: cryptorchidism incidence 30%, conception rate 50%, atrophy of seminiferous epithelium with few spermatogenic cells F2: cryptorchidism incidence 12.5%, conception rate 75% F3, F4: no cryptorchidism, conception rate 100%, normal sperm cells From F1 to F4: increased Dnmt levels, differentially methylated DNA sequences	Chen et al. 2015 [56]
	Orally, from E8 till E14 (500 mg/kg/day); SD rats	F1, F2, F3: testis, epididymal sperm (PN60)	F1 - F3: decreased sperm count, increased betaine levels, lowered expression of BHMT and global DNA hypomethylation	Yuan et al. 2017 [57]
Vinclozolin	Daily ip injections, from E8 till E14 (100 mg/kg/day); Hsd:SD rats	F1, F2, F3: testes at E16	F1, F2: changes in testis transcriptome, altered expression of methyltransferases F3: similar as F1 and F2, but most methyltransferases returned to the control generation levels	Anway et al. 2008 [58]
		F3: epididymal sperm	F3: differential DNA methylation in at least 16 promoter regions	Guerrero-Bosagna et al. 2010 [59]
		F3: pathologies of testis, seminal vesicle, prostate, liver, kidney, ovary, heart, ovary, uterus (PN120)	F3: unique tissue transcriptome, but common cellular pathways were identified between tissues; a number of identified gene clusters corresponded to the epimutations previously found in sperm that transmit epigenetic transgenerational inheritance of disease phenotypes	Skinner et al. 2012 [60]
		F3: fetal testis (E13 and E16)	F3: altered germ line transcriptome and epigenome, distinct in E13 germ cells (onset of gonadal sex determination) and E16 germ cells (after cord formation in the testis)	Skinner et al. 2013 [61]
		F3: testis and Sertoli cells (PN20)	F3: Increased spermatogenic cell apoptosis, 417 differentially expressed genes in Sertoli cells that have been linked with 22 pathways (incl. pyruvate/lactate metabolism pathway), > 100 promoter regions were differentially methylated in Sertoli cells	Guerrero-Bosagna et al. 2013 [62]
		F3: Sertoli cells (E13)	F3: altered SRY binding sites	Skinner et al. 2015 [63]
		F3: epididymal sperm (12 months)	F3: > 200 differentially expressed sncRNAs and associations with differentially methylated regions	Schuster et al. 2016 [64]
		F1, F3: epididymal sperm	F1: 290 altered DMRs F3: 981 altered DMRs No overlap between these DMR sets	Beck et al. 2017 [65]
		F1, F2, F3: pathologies (12 months) F3: epididymal sperm	F1, F2: few abnormalities F3: increased testis, prostate and kidney disease, changes in puberty onset in males, increased obesity rate in females; most of these diseases were linked to DMRs in sperm	Nilsson et al. 2018 [66]
		F1, F2, F3: epididymal sperm (12 months)	F1, F2, F3: altered DNA methylation and ncRNAs, distinct between direct *versus* transgenerational exposure F3: high numbers of differential histone retention sites	Ben Maamar et al., 2018 [67]
		F3: prostate (PN19-21 and 12 months)	F3: increased prostate abnormalities, changes in gene expression, ncRNA expression and DNA methylation	Klukovich et al. 2019 [68]
	Daily ip injections, from E7 till E13 (100 and 200 mg/kg/day); CD1 mice Daily ip injections, from E7 till E13 (100 mg/kg/day); inbred 129-mice (pathology analyses only)	F3: testis, prostate, kidney and ovary, epididymal sperm, isolated sperm heads (PN60-90 and 13–15 months)	F3: abnormalities in testis, prostate and kidney, polycystic ovarian disease, and spermatogenic cell defects (higher in low dose exposure than in high dose exposure); these effects were mainly seen in CD1 mice F3 (sperm heads of CD1 mice and lowest dose only): differential DNA methylated regions	Guerrero-Bosagna et al. 2012 [69]
	Daily ip injections, from E8 till E14 (100 mg/kg/day); *Big Blue* rats carrying *lacl* mutation-reporter transgene	F1, F3: kidney, epididymal sperm (< 1 year of age)	F1: no changes in mutation frequency in kidney and sperm F3: higher frequency of point mutations in kidney and sperm from control and in VCZ lineages, compared to F1; a subset of F3 animals showed a significantly higher mutation frequency in VCZ-exposed lineages, compared to F3 controls	McCarrey et al. 2016 [70]
	Daily ip injections, from E8 till E15 (100 mg/kg/day); Hsd:SD rats	F1, F2: testis (PN6) F1, F2: epididymal sperm, testis, prostate, seminal vesicle (13 weeks old)	F1, F2: no effect on spermatogenesis and fertility, no changes in methylation status	Inawaka et al. 2009 [71]
	Daily ip injections, from E10 till E18 (50 mg/kg/day); FVB/N mice	F1, F2, F3: epididymal sperm, tail, liver, skeletal muscle	F1: decreased DNA methylation at *H19* and *Gtl2* and increased DNA methylation at *Peg1*, *Snrpn*, *Peg3*; decreased motile sperm fraction F2, F3: the F1 effects decreased gradually	Stouder et al. 2010 [72]
	Orally, during pregnancy (1 and 100 mg/kg/day); CD1 mice	F1, F2, F3: testis (E13.5 and adult)	F1: male fertility rate reduces gradually by increasing dose, decreased number of PGCs, increased apoptosis in adult testis F2: fertility rate was recovered (in low dose lineage only), but still increments in apoptosis in adult testis of both high and low dose lineages F3: decreased fertility rate (both doses), recovery of number of PGCs (both doses), increased number of apoptotic cells in adult testis F1, F2, F3: deregulation of several microRNAs in PGCs	Brieno-Enriquez et al. 2015 [73]
	Daily ip injections, from E8 till E158 (1 mg/kg); SD rats	F1, F3: sperm, brain (hippocampal CA3 and central amygdala) (PN120)	F1, F3: hypermethylation, intergenic CpG islands proximal to pRNA were affected; fewer DMRs were found in brain compared to sperm, and in between tissue overlap of related genes was small	Gillette et al. 2018 [74]
EDC mixtures	Daily ip injections, from E8 till E14 (Permithrin: 150 mg/kg, DEET: 40 mg/kg, BPA: 50 mg/kg, DEHP: 750 mg/kg, DBP: 66 mg/kg, TCDD: 100 ng/kg, Jet fuel: 500 mg/kg); Hsd:SD rats	F1, F2, F3: blood, ovary, testis, epididymis, isolated sperm heads (PN90-120)	F3: plastics, dioxin and jet fuel were found to promote early-onset female puberty, decreased ovarian primordial follicle pool size, and spermatogenic cell apoptosis F3 (sperm heads): differential DNA methylated regions, specific to the exposure group	Manikkam et al. 2012 [75]
	Daily ip injections, from E8 till E14 (BPA 50 mg/kg, DEHP 750 mg/kg, DBP 66 mg/kg); Hsd:SD rats	F1, F3: kidney, ovary, uterus, testis, epididymis, prostate, seminal vesicle, (12 months) F3: epididymal sperm, isolated sperm heads	F1: increased kidney and prostate disease F3: Increased pubertal anomalies, testis disease, obesity, ovarian disease F3: differential DNA methylation regions in gene promoters of sperm	Manikkam et al. 2013 [76]
	Daily ip injections, from E8 till E158 (A1221: 1 mg/kg); SD rats	F1, F3: sperm, brain (hippocampal CA3 and central amygdala) (PN120)	F1, F3: hypermethylation, intergenic CpG islands proximal to pRNA were affected; fewer DMRs were found in brain compared to sperm, small overlap of related genes between sperm and brain	Gillette et al. 2018 [74]

## Results

### Search results and study selection

Our search strategy and identified numbers of records per level are presented in Fig. 1. Articles that included our three concepts—paternal, offspring, and epigenetics—resulted in 270 records. After screening titles and abstracts, we retrieved 43 articles that were relevant. Reasons for exclusion of 227 reports were cell culture studies (*n* = 2), studies in non-mammals (*n* = 20), no EDC exposure (e.g., name of chemical referred to methodology, nutrition, etc.) (*n* = 65), studies that solely described phenotypes or pathologies in offspring (*n* = 39), studies that did not explore epigenetic factors or where no inter- or transgenerational effects were explored (only individual effects) (*n* = 93), research or comments (*n* = 6), and reports written in Chinese (*n* = 2). Finally, we assessed the full text of 43 articles reporting on experiments where “fatherly exposures and offspring outcomes were scrutinized for epigenetic underlying mechanisms.” All studies were about research on animal models and none reported on human studies. Few animal studies were related to direct exposures in male animals, while most papers examined the paternal germ line through in-utero exposures. In brief, we found two papers on atrazine, three studies on benzo[a]pyrene (B[a]P), four on bisphenol A (BPA), eight on dioxins or dioxin-like compounds, four on DTT, two on phthalates, seventeen on vinclozin, and three on mixes of EDCs. In what follows, we discuss systematically selected articles by EDC, in alphabetical order. Study design and main findings are summarized in Table 1.

### Atrazine

Atrazine (ATZ) is a widely used herbicide, especially in corn and soy weed control. Hence, it is one of the most common contaminants found in underground waters in many countries [77–79]. In vertebrates, it has been shown to demasculinize and feminize male gonads [80]. Hao et al. examined its potential for transgenerational inheritance in a mouse model [35]. Pregnant mice were exposed via oral administration of ATZ between embryonic (E) day E6.5 and E15.5, and the male progeny was crossed for three generations with unexposed females. Control lineages started with mothers without ATZ exposure. Three biological replicates were used for each tissue studied in F3. The F3 generation adult males were studied for changes in somatic tissues (liver and hypothalamus) and testes. In the ATZ-treated lineage, the number of spermatozoa was reduced, meiotic defects were detected, and telomeres were often found to be fused during meiosis. Low levels of the acetylated histone H4K5ac—important in histone-to-protamine replacement—were detected and a decrease in protamine levels was reported. A transgenerational epigenetic inheritance was confirmed through the following observations. After a combined genome-wide ChIP-seq and RNA-seq approach in two generations (F1 and F3) of ATZ-exposed F0 animals, ATZ-derived males showed altered H3K4me3 peaks at the promoters of key pluripotency-associated genes. This was found in the F1 generation, as well as in testes of the F3 generation. Interestingly, changes in H3K4me3 occupancy in F1 sperm were often related to changes in RNA expression levels in non-testis tissues of F3 males [35]. These data suggest that histone modifications may be involved in the epigenetic inheritance phenomenon. Similar findings were found by McBirney et al., who investigated the potential inheritance of ATZ-induced toxic effects on the reproductive system of rats [36]. If exposed during in-utero development, the F1 offspring weighed less, compared to controls. The F2 generation was found to have increased frequency of testis disease and mammary tumors, early-onset puberty in males, and decreased body weight in females. Transgenerational F3 generation animals showed similar outcomes, including increased frequency of testis disease, early-onset puberty in females, motor hyperactivity, and a lean phenotype in males and females. More than half of the F3 males of the atrazine lineage had developed some abnormality or disease. Using a methylated DNA immunoprecipitation (MeDIP)-Seq protocol and bioinformatic analyses, differential DNA methylation regions (DMRs) were identified in sperm of all generations. Sperm DNA from F1 and F2 generation males of each condition (control and ATZ lineage) was pooled into three different pools. Each pool contained samples from five to 13 male rats. Sperm from the F3 generation was individually prepared and analyzed, because specific disease-associated biomarkers were explored in F3. The number of independent samples in the F3 generation was 50 for the treatment group and 18 for the control group. In conclusion, ATZ induced changes at the level of DMRs (also called “epimutations”) in all generations. Gene association analyses showed that these epimutations could be linked to some of the phenotypes found in F3, such as the lean phenotype and testis disease. Hence, the authors concluded that identification of specific epigenetic signatures in sperm can be used as a preconceptional diagnostic tool for future disease susceptibility in the offspring [36].

### Benzo[a]pyrene

Benzo[a]pyrene (B[a]P) is a polycyclic aromatic hydrocarbon found in grilled meats, coal tar, tobacco smoke, and wood-burning stoves.

Brevik et al. performed two in vitro fertilization (IVF) experiments using sperm cells from male mice, previously exposed to a single acute exposure to B[a]P [37, 38]. In a first study, they focused on microRNA (miRNA) expression levels in developing mouse embryos at 2-cell, 8-cell, and blastocyst stage, by paternal B[a]P exposure. The authors used sperm from six males (three exposed and three controls) and oocytes from 36 females. From each female, they fertilized one oocyte with sperm from an exposed animal and one oocyte with sperm from an unexposed animal. A total of 60 in vitro fertilized embryos were used for their analyses. At different stages of embryo development, specific miRNAs were found to be dysregulated by paternal B[a]P exposure. Most were involved in pathways related to metabolism, cell cycle, and cancer [38]. In a second study, they performed quantitative reverse transcription PCR (RT-qPCR) analysis of embryo lysates at various stages. A total of 184 in vitro fertilized embryos (derived from 92 B[a]P exposed and 92 control sperm) at five different developmental stages were used for their analyses. A panel of 78 mouse mRNAs was verified for expression in each embryo. They found that a large number of genes were differentially expressed in response to the paternal B[a]P exposure, in all stages. Particularly, a downregulation of several genes was observed at the blastocyst stage. By combining the two studies through an inverse correlation analysis (mRNA-miRNA pairs), followed by a search on the miRWalk database [81], they identified 37 genes responsive to 68 miRNAs selected from their first study [38]. Further functional analysis of these miRNAs and their targets revealed a number of biological processes triggered by paternal exposure to B[a]P, including DNA transcription, DNA damage response, cell cycle regulation, chromatin modification, oxidation-reduction processes, apoptosis, and embryo development [37]. Given that Brevik et al.’s studies involve father-embryo effects, they provide evidence for EDC-induced intergenerational epigenetic inheritance. Further investigation is needed to determine if offspring will inherit long-lasting health effects.

A similar approach was performed by Godschalk et al. [39]. They used six male mice chronically exposed to B[a]P (by oral gavage for 6 weeks) and six control males. This resulted in offspring with decreased mitochondrial function in the liver. More specifically, F1 male mice had a reduced activity of citrate synthase and β-hydroxyacil-CoA dehydrogenase. A potential epigenetic link for this male-specific result was detected through hepatic mRNA expression and miRNAs analyses. A seemingly controversial inverse association was found between downregulation of mitochondrial liver proteins and upregulation of related mRNAs. However, altered expression levels of miRNAs regulating the translation of these proteins could explain this discrepancy. Because mitochondria are known to induce oxidative stress, the authors assessed oxidative DNA damage by measuring 8-hydroxy-deoxyguanosine and malondialdehyde (MDA)-dG adducts. Both were decreased in liver of male offspring if their fathers had been exposed to B[a]P [39]. Again, this experiment is proof of an intergenerational effect after a paternal exposure to B[a]P.

### Bisphenol A

Bisphenol A is partly responsible for the current plastic pollution, because of its pivotal role in water bottle production, epoxy linings in food cans, etc. All reports selected on bisphenol A (BPA) exposure assessed potential transgenerational effects from oral administration, at least during gestation. Doses differed by study. Li et al. found in 2014 that maternal BPA exposure during gestation and lactation (GD0-PND21) disrupts glucose homeostasis in F2 offspring rats. BPA was given orally to ten pregnant dams, and compared to ten controls. Underlying epigenetic mechanisms were traced through investigation of genes and methylation status of promoter regions. The *Glucokinase* (*Gck*) promoter was hypermethylated and showed altered gene expression in liver of F2 rats previously treated with BPA [40]. Mao et al. provided further evidence—through the same experimental design—that dietary BPA exposure from gestation to lactation induces DNA hypermethylation of *Igf2* in pancreatic islets in the F2 generation (ten animals were used per group). Accordingly, F2 suffered from pancreatic β cell dysfunction and glucose intolerance. The authors explained this observation through methylation aberrancies in sperm, which they were able to measure in the F1 generation [41]. Similarly, Bansal et al. investigated how in-utero exposure to BPA alters functioning of pancreatic β cells and insulin levels across two generations in mice. Depending on the assay performed, three to six litters were tested per group. In general, one mouse per litter was randomly selected for each assay. A high and a low concentration of BPA was used, both representative of human exposure levels. Both doses resulted in a male-specific effect on insulin secretion in F1 and F2 offspring, and immune responses were perturbed in pancreatic islets of both generations. A dose-specific effect was observed in expression of genes important in inflammation and mitochondrial function, until the second generation. An increased *Igf2* expression persisted in pancreatic islets of F1 and F2 male offspring, which was also associated with altered DNA methylation. Hence, again an intergenerational risk for impaired insulin secretion and diabetes could be assessed and linked to ancestral BPA exposure [42]. Drobná et al. investigated brain tissues in the F3 generation and explored which genes are potentially transgenerationally affected by ancestral exposure to BPA [43]. After dietary administration of BPA during F0 gestation, brain of F3 juvenile males were investigated (three per group) and RNASeq showed that the *Meg3* gene was involved in the inherited behavioral anomalies. Upregulation of *Meg3* expression was suggested to play a role in the brain-pituitary-adrenal axis. However, DNA methylation analysis of *Meg3* DMR did not show a link with ancestral BPA exposure or its altered mRNA levels [43]. Note, this was evaluated in brain, but not in sperm.

### Dichlorodiphenyltrichloroethane

Dichlorodiphenyltrichloroethane (DDT) is a pesticide that has been used worldwide since the 1940s. Although banned by many countries, nearly 5000 tons per year is still being used worldwide, mainly to control vectors for malaria and visceral leishmaniasis. India, China, and African countries are the largest consumers [82, 83]. Toxic effects of direct exposure to DDT in humans include reproductive disease, neurological disease, developmental abnormalities, and cancer [44]. Exposure to DDT and its breakdown product dichlorodiphenyldichloroethylene (DDE) has also been associated with adverse health outcomes such as overweight in children [84].

Skinner et al. investigated the potential transgenerational actions of two concentrations of DDT on obesity and associated disorders, from F1 till F4 (eight litters were used per lineage) [44]. Doses of DDT used were comparable to human environmental exposure levels. No obese offspring were born after daily intraperitoneal (ip) injections in pregnant rodents (from E8 till E14). However, signs of obesity only appeared in the F3 generation. Additionally, other transgenerational diseases were observed in F3, including testis disease, polycystic ovarian disease, immune abnormalities, and kidney disease. Using a MeDIP procedure, followed by a promoter tiling array chip (MeDIP-chip), sperm DNA was analyzed for methylation outcomes in sperm from F3 offspring. Numerous DMRs were found to be affected. The authors excluded those that were also positive in other studies (by exposure to other EDCs). This resulted in 28 DMRs that were significantly different and specific to DDT exposure. It needs to be noted that DDT and its metabolites have similar chemical structures to BPA derivatives [83]. Hence, it might be that by using this approach, some effects may have been missed. Further bioinformatic analysis of genes associated with these 28 DDT affected sperm DMRs identified a number of genes known to be involved in obesity or polycystic ovarian disease [44]. A comparison of DDT-induced alterations in sperm DMRs demonstrated unique changes in the F1, F2, and F3 generations.

Based on the same exposure protocol and animal model, a following study explored other components of the epigenetic machinery, such as non-coding RNAs (ncRNAs) and histone retention in purified cauda epididymal sperm [45, 46]. Sperm pools of several rats were used to obtain sufficient amount of RNA or DNA. Through RNA-Seq and bioinformatic analyses, the group of Skinner detected differential levels of ncRNAs in sperm from DDT lineages versus controls. Each generation had unique expression levels of small non-coding RNAs (sncRNAs) and long non-coding RNAs (lncRNAs). One particular class of sncRNAs, piRNAs, represented the highest number observed after ancestral DDT exposure, followed by small tRNAs. Most lncRNAs were found in the F1 and F3 generation, while the F2 generation counted fewer lncRNAs. In contrast to DMRs and ncRNAs, the F1 and F2 generation sperm (directly exposed) did not show altered histone retention, while new histone H3 retention sites were identified in the transgenerational F3 generation sperm [46]. The majority of these differential histone retention sites (DHRs) were found to be intergenic, suggesting a role in regulation of ncRNAs. Phenotypic observations were limited to measurements of cellular apoptosis in rat testes. The F3 generation DDT lineage showed the highest level of spermatogenic cell apoptosis, supporting a transgenerational phenotype of the DDT model used [45]. In a subsequent study, the same research group tried to better understand epigenetic changes at the level of DMRs caused by ancestral DDT exposure. They focused their research on transgenerational (F3) effects in male germ cells at different developmental stages and compared three pools per condition (each pool contained sperm from three to seven males); F3 animals from exposed lineages were compared to control lineages [47]. They isolated embryonic day-16 (E16) prospermatogonia, postnatal day-10 (P10) spermatogonia, adult pachytene spermatocytes, round spermatids, caput epididymal spermatozoa, and caudal sperm from DDT lineage F3 generation rats. Although the authors point to a “cascade of epigenetic alterations” initiated by earlier DDT exposure to primordial germ cells (PGCs), no measurements were performed on the F1 and F2 generations. Hence, insights in intermediate epigenetic effects remain scarce. Nevertheless, the authors expanded their analyses and performed gene association studies to predict potential transgenerational health effects. They found that metabolic pathways and pathways common in cancer were associated with DMRs aberrances in nearly all stages of sperm development. Most recently, King et al. measured the incidence of adult-onset pathologies in F1, F2, and F3 generations, linked to ancestral DDT exposure. Identified pathologies included late-onset puberty, prostate disease, kidney disease, testis disease, and obesity [85]. Future studies are needed to further confirm these interesting disease correlations.

### Dioxin and dioxin-like compounds

The term “dioxins” refers to a group of chlorinated organic compounds that have two benzene rings in its structure connected by two oxygen atoms. Most dioxins are formed due to human activities, such as household trash burning, incineration of plastics, emission of automobiles, pesticide production, etc. Because of their lipophilic characteristics, they dissolve readily in fatty compounds and bio-accumulate in the food chain. Hence, the major source of human exposure to dioxins is through diet, via consumption of meat, fish, and dairy products. While it is known that short-term and long-term exposures to dioxins affect human health, they can also transfer prenatally through the placenta, resulting in development of chronic diseases in later life [86]. However, influences through the paternal line are less documented.

#### 2,3,7,8-Tetrachlorodibenzo-p-dioxin

The most toxic dioxin known is 2,3,7,8-tetrachlorodibenzo-p-dioxin (TCDD). It was one of the main contaminants of Agent Orange, a herbicide sprayed widely in the Vietnam war [87]. Nowadays, it is formed during incineration processes of waste, in paper and pulp bleaching, and through emissions from steel foundries and motor vehicles [88].

Manikkam et al. examined if oral administration of TCDD promotes transgenerational inheritance of diseases. They found that a dose of 0.1% of the median know lethal dose (LD50) was able to promote transgenerational epigenetic effects or some diseases [48]. Kidney diseases were detected in the F3 generation TCDD lineage males. A decrease in spermatogenetic cell apoptosis was limited to the F1 generation only. But, no testis diseases, changes in sperm number, or motility were observed in F1 or F3 generation rats. F3 males demonstrated a TCDD-induced change in DNA methylation at 50 DMRs in sperm [48]. In order to perform these experiments, they used nine F3 generation rats per condition (TCDD or control), and pooled sperm from three animals to obtain enough DNA. Unfortunately, no epigenetic analyses on sperm were performed in the F1 and F2 generation. Hence, intermediate epigenetic effects remain unknown. Ding et al. conducted a global methylation analysis of late pregnancy mice placentae (E18.5) after TCDD exposure in the F0 generation [50]. They examined multiple placentae per litter, using at least five litters per group. This revealed more than 2000 differentially methylated CpG regions. Most corresponded to promoters of genes where aberrancies have been associated with preterm birth, in mice but also in human. Ingenuity pathway analysis was used to predict the affected biological pathways. This revealed that *Esr1* was one of the main upstream regulators to be impacted by TCDD exposure. This gene is known to modulate progesterone receptor (*Pgr*) and insulin-like growth factor (*Igf2*) gene expression. Validation of methylation status and expression of *Pgr* and *Igf2* in F1/F3 sperm and F3-derived placentae showed consistent findings, although results did not always reach statistical significance [50]. It should be noted that the limited transgenerational effects measured could be due to selection, given that the most severely impacted F1 mice exhibited complete infertility. Nevertheless, after further investigation of placental samples for *Pgr* and *Igf2* genes products, such as mRNAs and protein expression, reduced profiles were detected in F1 and F3 male-derived samples, compared to control placentae. This was in line with a significant increase of *Dnmt1* mRNA in placentae, normally suppressed by progesterone and estradiol [50]. The study of Ding et al. focused on genes involved in preterm birth and placental dysfunction. Hence, other pathways or genes were not explored any further.

Ma et al. evaluated transgenerational effects of ancestral TCDD exposure at two DMRs of *IGF2*/*H19* in rat F1 and F3 generation liver tissues. CpGs that are part of the imprinting regulatory control region (ICR) of *IGF2* were hypermethylated. And, DMRs located upstream of the neighboring non-coding H19 were hypomethylated, compared to control animals. These opposite epigenetic effects were present in both generations (F1 and F3). The authors attributed these TCDD-induced differences in response to the aberrant expression patterns of DNA methyltransferases they equally observed (DNMT1, DNMT3A, and DNMT3B) in treated animal lines [49]. DNA methyltransferases are highly conserved enzymes that establish and maintain methylation marks.

#### 1,1-dichloro-2,2-bis(4-chlorophenyl)ethene

Song, Yang, and colleagues studied in-utero exposure to 1,1-dichloro-2,2-bis(4-chlorophenyl)ethene (p,p′-DDE) in pregnant rats [51–53]. Their work showed that p,p′-DDE induced a transgenerational inheritance of impaired spermatogenesis with altered epigenetic modifications at the level of DMRs at imprinted genes, including *IGF2*/*H19* and *Gtl2* hypomethylation in all generations (*n* = 3 per group) [51, 53]. Consistently, genes were upregulated in sperm and testis of three generations [53]. Other observations included impaired glucose tolerance, abnormal insulin secretion, and β-cell dysfunction. This was also observed until the F3 generation in male lineages [52].

The authors further explored potential involvement of DNMTs and compared methyltransferases in embryonic testes of the three generations. They measured only decreased levels of DNMT1 and 3a in the F1 and F2 generation, and no changes in the F3 generation (versus control samples). From these observations, the authors concluded that inheritance initially (from F0 to F2) involves the DNA methylation machinery via DNA methyltransferases. But then (from F2 to F3), the environmental signature appears to be transmitted through other yet unknown (epigenetic) mechanisms [53].

#### Methoxychlor

A paper by Anway et al. suggested as early as 2006 that methoxychlor (MXC) can promote a transgenerational disease state. However, the molecular mechanisms had not been explored yet [89]. About 10 years after this report, the same research group examined how MXC causes transgenerational inheritance of adult-onset disease in rats and how DNA methylation can be affected in the germ line [55]. After exposure to MXC during fetal gonadal development (E8 to E14), several characteristics were examined in the F1 and F2 generations (*n* = 8 litters per lineage), including body weight, puberty-onset, and histology of testis, prostate, ovary, uterus, and kidney. No major toxicity and endocrine effects were found, but an increased incidence of kidney disease (in females), ovary diseases, and obesity were observed. Especially the F3 generation seemed to have the highest risk of comorbidities. MeDIP followed by a tiling array chip was performed to allow sperm DNA methylation mapping across the rat genome in three generations. Thirty-seven differentially methylated regions were detected in MXC lineage rats, compared to control lineage rats. These “epimutations” were confirmed through a stringent approach of three different experiments. The authors further scrutinized a potential sex-specific transmission of diseases from the F3 to F4 generation. This revealed inheritance of male obesity, male and female kidney disease and transmission of multiple diseases through the female germ line [55]. Still, several knowledge gaps need to be addressed. For instance, it is not clear how epimutations found in F3 sperm can be translated into aberrances in the F4 and the next generations. No data on the intermediate (F2) generation were reported. Furthermore, research on oocytes, which could lead to a better understanding of disease inheritance through the female germ line, is missing.

Stouder et al. investigated deleterious effects of a MXC [54]. Adult male mice treated with MXC resulted in sperm with decreased percentages of DNA methylation *Meg3* CpGs and increased DNA methylation at *Mest*, *Snrpn*, and *Peg3* CpGs. In-utero exposure during the embryonic time window from E10 until E18—or until the moment that imprinting is expected to be reset in prospermatogonia—resulted in lower sperm concentrations in the F1 generation (*n* = 9–10). Sperm DNA from F1 showed a decrease in methylation at the *H19* and *Meg3* CpGs, and a slight increase was measured at the *Mest*, *Peg3*, and *Snrpn* CpGs. Interestingly, these effects were still present and highly significant in F2 offspring sperm, but transgenerational effects (in F3, *n* = 9–10) were limited to only a few genes, *H19* and *Peg3*. Moreover, a tendency to evolve to normal values was observed [54].

### Phthalates

Phthalates belong to the most widely used plasticizers. Their application ranges from food packaging materials to their use in medical devices. Epidemiological studies have shown that exposure to several phthalates are linked to impaired male reproductive function and semen quality [90, 91].

Chen et al. studied the effects of exposure to bis(2-ethylhexyl)phthalate (DEHP) during the critical embryonic period of rat (E7-E19) through gavage [56]. DEHP exposure modified DNA methylation in the first generation (F1) and deteriorated reproductive function. Sperm deformities and cryptorchidism were observed, which was associated with raised Dnmt levels (Dnmt1, Dnmt3a, Dnmt3b). A MeDIP-seq analysis revealed several differentially methylated DNA sequences between F1 and F4 generation (*n* = 8 per generation), suggesting different mechanistic processes in directly and indirectly exposed rats. Evaluation of phenotypes such as mating potential in DEHP-treated lineages resulted in conception rates of 50%, 75%, and 100%, in F1, F2, and F3/F4, respectively. Hence, complete recovery was observed in the F3 and F4 generations. Another study in rats, by Yuan et al., indicated persistent epigenetic effects after phalate exposure. Peroral dibutyl phthalate (DBP) exposure of pregnant rats (*n* = 5) from E8 until E14 reduced the number of sperm and Sertoli cells and serum concentrations of testosterone in at least three generations. These changes in male reproductive function were associated with distinct changes in DNA methylation. Hypomethylation at the promoter of the *Fstl3* gene until the F3 generation could explain failure of normal spermatogenesis. Interestingly, metabolic analysis revealed an increased level of betaine and a decreased level of betaine homocysteine S-methyltransferase (BHMT) in testes of both in F1 and F3 generation offspring. This suggests a disturbed methionine cycle that could explain failure of transgenerational spermatogenesis [57].

### Vinclozolin

Vinclozolin (VCZ) is an anti-androgenic fungicide used in growing many vegetables and fruits. Animal models have shown that VCZ exposure around the time of embryonic sex determination causes a reduction in spermatogenetic capacity in offspring [89]. Testes phenotypes can be transgenerationally transmitted for at least four generations; suggesting an important role of the epigenetic machinery [92]. Most studies on VCZ-induced epigenetic transgenerational inheritance phenomena have been performed by Skinner’s research group. Below, we first discuss Skinner’s papers, followed by few results from other groups.

#### Results by Skinner’s group

The group of Skinner exposed F0 pregnant rats during gonadal sex determination (E8-E14) to VCZ through intraperitoneal injections. At least eight lines were used for VCZ exposure, and eight lines were generated for controls. More than 196 genes were found to be differentially expressed in testis of F1–F3 generations, compared to non-exposed lineages [58]. Next, DNA methyltransferases (Dnmts; mainly Dnmt3A and Dnmt3L) and euchromatic histone methyltransferase (Ehmt1) were decreased in embryonic (E16) testis of the F1 and F2 vinclozolin generation, compared to controls. Changes of most methyltransferases appeared to be returning to normal control levels in the F3 generation, except for Ehmt1. Differences in responses to direct versus indirect exposure to VCZ suggest that alternative (yet unknown) mechanisms may be activated [58]. It should be noted that whole testes tissues were used in this experimental model. Hence, samples comprised several stages of spermatogenic development, as well as somatic cells. Next, sperm isolated from the cauda epididymis of the third rat generation (*n* = 3 per group) was scrutinized, after ancestral exposure to VCZ. At least 16 different promoters were found to be differentially methylated [59]. Gene co-expression network analyses of somatic tissues in F3 showed that these sperm epimutations (detected in F3) corresponded to specific gene clusters important in adult-onset of diseases (this was verified in six male and six female rats of F3) [60]. Several years later, they implemented a protocol involving MeDIP followed by next-generation sequencing and further scrutinized the effects of VCZ on potential epimutations in several generations [65]. VCZ exposure resulted in about 290 DMRs that were changed in F1 sperm, and 981 DMRs were altered in the F3 generation sperm. No overlap between these DMR sets could be observed, suggesting that distinct epigenetic mechanisms are triggered in direct effects compared to transgenerational effects [65]. Skinner et al. also studied potentially associated inheritance of disease pathways. They isolated sperm at two different developmental stages from F3 rats [61]. Primordial germ cells (PGCs) showed altered DNA methylation and transcriptome profiles, compared to controls. Differentially expressed genes were dramatically different in PGCs isolated at embryonic day 13 (E13) versus day 16 (E16); 592 genes versus 148 genes, respectively. Again, limited overlap was seen between these two sets of genes. Only 25 differentially expressed genes were similar. Pathway analysis showed that nearly 20 pathways were altered at E13, and only one pathway seemed to be disturbed at E16. Notably, one pathway that was prominently affected at E13 was the olfactory transduction pathway [61]; an extremely important system in rodents. Similar experiments in mice, where the F0 generation was exposed to a BPA-containing diet, showed that olfactory discrimination or social recognition was affected in the next generations [93]. Although the latter study did not investigate potentially involved epigenetic mechanisms (hence, it was not included in our selected articles), it indicates that EDCs induce epigenetic signatures through the male germ line contributing to behavioral aberrancies in future offspring.

In another report where six pregnant females were treated with ip VCZ injections, fertility-related outcomes in F3 males were studied together with epigenetic responses [62]. Guerrero-Bosagna et al. linked transgenerational increased apoptosis in sperm cells of VCZ lineage males to altered gene expression in supporting Sertoli cells. The F3 generation of Sertoli cells (pooled from three times 2–6 males per condition) counted 417 genes that were differentially expressed in the VCZ lineage, compared to controls. Among the 22 regulatory pathways identified, at least one key pathway important in pyruvate/lactate production showed a direct mechanistic link with induction of apoptosis in sperm cells [62]. Next, about 100 promoter regions were differentially methylated in F3 Sertoli cells. These epigenetic alterations were associated with genes known to be involved in male infertility disorders. In a following study, Sertoli cells were investigated at an earlier stage. Results of this approach was published a few years later and increased our understanding of the molecular mechanisms involved in VCZ-induced epigenetic transgenerational inheritance of testis disorders [63]. In this report, Skinner and his team investigated transgenerational changes in the transcription factor, SRY (or “sex determining region on the Y chromosome”), during early stages of Sertoli cell differentiation. They identified multiple altered SRY binding sites in Sertoli cells of fetal gonads in the F3 generation of VCZ-treated lineages [63]. The fact that these sites are already affected in the fetal testis could explain the observed transgenerational epigenetic alterations in adult Sertoli cells [62].

Schuster et al. undertook an alternative mechanism such as sncRNA consisting mainly of miRNAs, tsRNAs, mitochondrial genome-encoded small RNAs (mitosRNAs), and piRNAs to assess their role in transgenerational inheritance [64]. They checked for alteration in sncRNA in sperm between F3 generation vinclozolin lineage rats and control (*n* = 3 pools of three males for each group), and observed over 200 differentially expressed sncRNAs, with tRNAs being greatly affected [64]. Gene targeting prediction analysis showed correlations with earlier described and published phenotypes, such as apoptosis [62], and brain/behavioral problems [74, 94]. Maamar et al. extended these analyses and investigated all generations for several epigenetic factors, including DNA methylation, ncRNAs, and histone retention in sperm [67]. DNA methylation and ncRNAs were altered in sperm of all three generations, but directly exposed generations (F1 and F2) had distinct epimutations compared to the indirectly exposed generation (F3). High numbers of differential histone retention sites were found in sperm from the F3 generation, which was not seen in F1 and F2 sperm [67]. Hence again, these results corresponded to other publications by Skinner’s group, where a general trend of epigenetically affected progeny was seen for several generations, but epigenetic alterations were different when comparing inter- versus transgenerational effects. In order to identify specific biomarkers, Nilsson et al. correlated epigenetic findings in F3 sperm with inherited health issues in this rat model. Specific differential DMRs in sperm could be linked with testis, prostate, and kidney diseases [66]. Klukovich et al. further analyzed prostate epithelial and stromal cells of young F3 rats (*n* = 3 pools of a total of 22–30 males, per condition), corresponding to the end of weaning and prior to the onset of potential pathologies. Their study confirmed that ancestral VCZ exposure influences the sperm epigenome and development of prostate diseases in the F3 generation [68]. Skinner and colleagues repeated part of their experiments in a mouse model [69]. They exposed F0 gestating females to VCZ through daily intraperitoneal injection during gonadal sex determination (E7–E13). This produced F3 mice with higher incidence of spermatogenic cell defects, testicular abnormalities, prostate abnormalities, kidney abnormalities, and polycystic ovarian disease. A comparative MeDIP-chip analysis on sperm of CD1 outbred animals (*n* = 6) showed that at least 40 DMRs had transgenerationally altered DNA methylation patterns [69]. However, the authors commented that none of the gene promoters found in mouse corresponded to the gene promoters earlier identified in rat [59]. Although in both models the F3 generation was explored, cells were isolated in a slightly different way. In their rat model, spermatozoa were generally isolated from the cauda epididymis while in their mouse model, isolated sperm heads were used.

A critical research paper by McCarrey and Skinner’s group further investigated if VCZ-induced epigenetic transgenerational effects were caused by the formation of genetic mutations that impact epigenetic programming (called “secondary epimutations”) or by epigenetic inheritance without any genetic change (called “primary epimutations”) [70]. Using a *lacI* mutation-reporter transgene rat model, they measured frequency of mutations in kidney tissue and sperm from F1 and F3 (the number of samples tested varied between 4 and 10). They found no difference in mutation frequency in the F1 generation between VCZ- and control-lineage offspring, confirming that earlier findings were caused by primary epigenetic effects. Surprisingly, when investigating mutation frequencies in F3 generation VCZ- versus control-lineage descendants, an elevated frequency of point mutations was found in a subset of tissues evaluated (three out of eight in sperm and three out of eight in kidney). The authors defined this type of inheritance “tertiary epimutations.” This new finding opened new perspectives on the phenomenon of inheritance: in-utero exposure to VCZ initially induces primary epimutations, but later on—in subsequent generations—it promotes elevated accumulation of point mutations in some descendants, resulting in transgenerationally inherited phenotypes [70].

#### Results by other research groups

Few other research groups have studied transgenerational effects of VCZ, but results are somewhat conflicting. For instance, Inawaka et al. could not find abnormalities in spermatogenesis or testicular phenotypes in the F1 generation after in-utero exposure to VCZ (at least five males were used per condition) [71]. They further focused on one specific gene that was found by Anway et al. to be hypomethylated after VCZ exposure, namely the *LPLase* gene. This gene codes the lysophospholipase enzyme, important in a number of physiochemical and biochemical modifications of the plasma membrane of spermatozoa before fertilization. The idea was that if expression of *LPLase* is affected and inherited, anomalies would be detectable in the F1 and F2 generations of male. However, Inawaka et al. did not find any alterations of DNA methylation at exon 14 of the *LPLase* gene, which was the same site studied by Anway et al. [89]. Notably, the study by Inawaka et al. did not measure expression levels of this gene to further explore and confirm potential discrepancies.

The following research groups found some long-term effects from early VCZ exposure. After intraperitoneal administration of VCZ in pregnant mice, from E10 till E18, Stouder and Paoloni-Giacobino studied a handful of DMRs in motile sperm fractions by pyrosequencing [72]. The F1 generation showed hypomethylation at paternally imprinted genes (*H19* and *Gtl2*) and hypermethylation at maternally imprinted genes (*Mest*, *Snrpn*, and *Peg3*). The effects of VCZ were transgenerational, but they disappeared gradually from F1 to F3 (*n* = 8–15 mice). In somatic cells, DNA methylation was altered at the *Peg3* gene, which persisted in F2 and F3 [72]. Brieno-Enriquez et al. investigated a diet-based effect of VCZ during pregnancy of mice, using low dose, high dose, and controls (*n* = 20 mothers per group). They analyzed reproductive phenotypes and miRNA expression levels of purified PGCs by real-time qPCR. First, a reduction in number of embryonic PGCs was seen in F1 and F2 generations, but a recovery was found in F3 [73]. Higher rates of apoptotic cells and decreased fertility indexes were also observed in adult testes, but no dose-response effects could be defined. Next, deregulation of several miRNAs was found. In brief, a transgenerational upregulation of *let-7* miRNA and *miR-23b* in PGCs of VCZ lineage male mice was associated with a downregulation of some key regulatory genes in PGC development, such as *Lin28* and *Blimp1*, along three generations. Hence, these data provided evidence for a transgenerational VCZ-induced deregulation of expression of microRNAs important in germ cell differentiation [73]. Most recently, Gillette et al. demonstrated that exposure to a low dose of VCZ in rats during gestation (from E8 till E18) causes a transgenerational aberrant effect at the level of DMRs in sperm and two nuclei of the brain (hippocampal CA3 and central amygdala) [74]. They scrutinized the male breeding line, using males (F1; *n* = 4 litters) from exposed dams to create F2 (*n* = 8 litters). Consequently, F2 males were used to produce F3 litters. Directly exposed (F1) and ancestrally exposed (F3) males were analyzed for potential epigenetic outcomes. Overall hypermethylation was seen in F1 and F3 sperm (*n* = 8), in response to VCZ. Interestingly, several intergenic CpG islands proximal to promotor-associated RNA (pRNA) regions were differentially methylated, in F1 and F3. This was also seen if 2,3′,4,4′,5-pentachlorobiphenyl (PCB) mixtures were used (see section on EDC mixtures) [74]. These findings suggest that this class of non-coding RNAs belong to an important core of EDC-targeted regions inducing epigenetic inheritance through several generations. Brain tissues of F1 and F3 showed an equivalent number of DNA methylation alterations. A comparison between changes in F3 sperm *versus* brain demonstrated a small overlap [74].

### EDC mixtures

As discussed above, the study of Gillette et al. measured DNA methylation changes in rat F1 and F3 generations after exposure to VCZ. The authors also measured epigenetic effects of Aroclor 1221 (A1221), a commercially available pesticide containing a mixture of polychlorinated biphenyls (PCBs) with known estrogenic modes of action [74]. Transgenerational inheritance of phenotypic effects from exposure to A1211 has been reported by others [95]; these effects were not always observed in the directly exposed F1 offspring, but emerged in F2 and F3 generations. Proof of epigenetic involvement has been demonstrated by Gillette et al. A1221-induced aberrances in the epigenome of sperm and brain were measured in at least three generations. The authors suggested that patterns of hypermethylation at CpG islands in sperm could induce genomic instability, which in turn could increase the risk for spontaneous mutations. This phenomenon fits the “tertiary epimutation” theory on how diseases can be passed on to future generations [70, 74]. Two subsequent studies by Manikkam et al. in a rat model confirmed that in-utero exposure to mixtures of EDCs—as is the case in our daily living environment—promotes transgenerational epigenetic inheritance of adult-onset diseases [75, 76]. In 2012, they showed that in-utero exposure to plastic mixtures (bisphenol A and phthalates), pesticide mixtures (permethrin and insect repellant DEET), dioxin (TCDD), or a hydrocarbon mixture (jet fuel, JP8) causes early-puberty onset in females and decreased gonadal function in females and males [75]. Sperm heads of F3 were examined via MeDIP-chip assay analysis (*n* = 3 pools of three animals each, per exposure group). Distinct epigenetic patterns in DMRs were found in relation to these ancestral environmental exposures. Each exposure showed distinct epigenetic signatures, which opens opportunities to use these as future biomarkers. In 2013, the same group reported histological abnormalities in F1 and F3 progeny after F0 exposure to EDC mixtures, such as from plastics. A mixture of BPA, DEHP, and DBP was given to female rats during E8–E14 [76]. Outcomes included pubertal abnormalities, testis and prostate diseases, polycystic ovarian disease, primary ovarian insufficiency, and obesity. This was tested in a F3 generation of 40 indirectly exposed animals versus 56 control animals. If the exposure dose was halved (*n* = 58 animals), plastics did not significantly affect prostate and kidney in the third generation (F3). An association analysis of combined data of their earlier results in sperm [75], and results on tissue characteristics of F3 rats [76], revealed a number of DMR-associated genes in F3 sperm correlated to the diseases observed [76]. Unfortunately, dose-response analyses were limited to two doses only. And, although the window of exposure was well chosen, another window is needed as a comparison. Only then can the gonadal sex determination period be defined as being the most susceptible period for transgenerational damage by EDCs. Next, the F2 generation was not studied, which prevents a thorough examination of this (male) line of epigenetic inheritance. However, it should be noted that it is unlikely that this second generation would show conflicting results.

## Conclusions

### Main messages from the current literature on epigenetic inheritance of ancestral EDC exposure

EDCs are important environmental pollutants found everywhere in our daily lives and foods. They are able to act as hormone receptor mediators and thus dysregulate homeostatic mechanisms, reproduction, and development [96, 97]. However, EDCs not only disrupt the exposed individual, other endpoints include subsequent generations. Through a systematic search of the current literature, we found 43 EDC-related studies where epigenetic signals in progeny were validated for harmful health effects, particularly through the paternal line of inheritance. Our selected reports included the most commonly used and characterized synthetic EDCs, such as pesticides (e.g., DDT, MXC, ATZ, and VCZ), plasticizers (BPA and phthalates), B[a]P, and other compounds of industrial origin. A remarkable observation was that these exposure-oriented studies were only performed in animal models. No human studies were found. In a few studies, offspring was studied after male rodents were preconceptionally exposed to EDCs during adulthood. All remaining studies involved pregnant animals that were exposed to the pollutant of interest. Indeed, in-utero development is an important “window of susceptibility” to environmental influences, because gonads are being developed and the epigenome is still malleable and able to receive and store signatures originating from the maternal environment. However, this mother-to-child point of view limits our ability to further explore other potential windows such as through the father’s environment before conception [13]. Especially in respect of public health applications, it is important to understand how exposures or experiences throughout adult life of potential future fathers influence his progeny.

The majority of the studies selected in this review provided clear proof of epigenetic effects through the male germ line. Hence, the available evidence strongly indicates that EDCs can increase the risk for chronic diseases in offspring from earlier exposed (great)grandparents. Changes in the epigenome were mostly linked with fertility-related disorders or several other adult-onset diseases. Aberrant phenotypes and epigenetic marks were frequently found until the third generation. However, not all studies could fully confirm this transgenerational epigenetic effect. Some reports only verified intergenerational epigenetic effects from EDC exposure. First, after paternal exposure to B[a]P, only the embryos were studied [37, 38]. Second, in the case of early exposure to phthalates—known for nearly 20 years to affect testis in immature males [98]—results were conflicting. Chen et al. concluded that phthalate-induced transgenerational phenotypic and epigenetic modifications gradually diminished after several generations [56] while Yuang et al. provided evidence for a maintained metabolic effect and DNA methylation shifts until the third generation [57]. In-utero exposure to methoxychlor in mice did not alter DNA methylation at imprinted genes of the F3 generation, while it did affect DNA methylation in directly exposed animals [54]. In a similar animal model, VCZ affected DNA methylation at specific imprinted genes, but these aberrancies gradually disappeared at most genes from F1 to F3. Yet, DNA methylation at the imprinted *Peg3* gene remained significantly low in sperm of the F3 generation. Exploration of somatic cells showed that *Peg3* was also aberrantly methylated in the F2 and F3 generations [72]. *Peg3* encodes a zinc finger protein important in multiple cellular processes. Aberrances in its expression have been linked to an affected reproductive health and disturbed metabolic homoeostasis [99]. Future experiments with various windows of exposures—and where both the maternal and the paternal line are explored—may shed more light on these processes. A study in rodents by Drobna et al. found alterations in expression levels of imprinted genes in brain if the ancestral generation had been exposed to BPA, but this study lacked an exploration of epigenetic effects at the level of imprinted genes in male germ cells [43]. It has been documented that BPA also affects the brain in humans, causing neurobehavioral problems. Although no underlying cause of a possible environmental exposure was reported, an epidemiological study by Fuemmeler et al. showed that modification of inherited imprint regulatory regions in offspring cord blood is related to childhood behavioral problems [100]. Notably, human data also showed aberrances at the level of imprinted genes if men were exposed to low-doses of organophosphates [15]. Although not yet confirmed, we assume that DNA methylation patterns at imprinted genes in sperm could be used as biomarkers of earlier exposure to EDCs.

Converging results of human and animal data from different EDC exposures suggest that the same network of genes orchestrating specific cellular pathways may be affected. However, it is still unclear how EDCs are able to target genes (such as *Peg3*) in somatic cells of an individual that never experienced the exposure. Furthermore, it is also unclear whether this observation in a third generation would also occur in humans, which obviously remains a difficult issue to solve. Remarkably, comparison of different animal models (e.g., mice versus rat) shows that EDC-induced epigenetic effects are not always identical [59, 69]. Next, variations in epigenetic responses at all levels (DNA methylation, regulatory enzymes, and ncRNA expression) were found among different generations [52, 54, 58, 65, 67]. For instance, after VCZ exposure in F0 rat, methyltransferases were differentially expressed in the F2 versus F3 generation [58]. Although, the same authors earlier reported that adult-onset diseases could be observed in up to four further generations [92]. A reasonable hypothesis that could explain potential discrepancies in epigenetic effects between generations was proposed by McCarrey and Skinner. They launched the idea of “tertiary epimutations,” where transgenerational inheritance of diseases can be explained by a combination of epigenetic events, followed by an accelerated rate of genetic mutations or increased genomic instability [70]. We hypothesize that this could also explain why the Hao et al. study found that H3K4me3 was highly present in sperm and tissues of mice progeny after (grand)mothers were exposed to atrazine [35]. H3K4me3 is known to be present at sites of DNA damage (double-strand breaks) where it is involved in DNA repair and maintenance of genome stability [101]. Hence, modifications of H3K4me3 in the F3 generation of ATZ-treated F0 mice (as was detected by Hao et al.) might be due to a DNA damage response, instead of a pure epigenetic change. Recent studies in *Caenorhabditis elegan*s also showed that chronic exposures to environmental pollutants cause an increased number of mutations, deletions, and insertions, even ten generations further. Intermediate epigenetic mechanisms were suggested, but the exact mechanisms have not been explored yet [102].

**Fig. 2:**
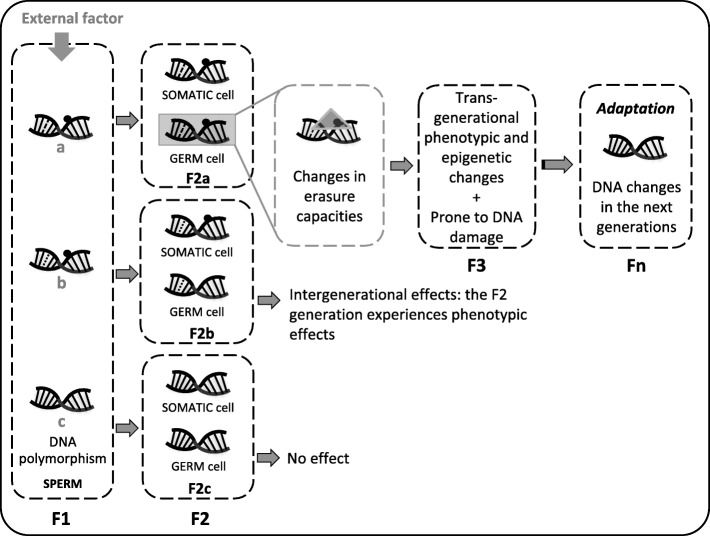
Hypothesis on the interplay between genetics and epigenetics in response to ancestral exposure to EDCs. Direct exposure to F1 (through pregnancy of F0 or directly in the life course of F1) causes a “temporal” or a “persistent” epigenetic effect (represented by a black dot), or no effect at all. A temporal effect relates to an intergenerational transmission of the exposure, and a persistent effect refers to a transgenerational phenomenon, as earlier described by Skinner [32]. We here suggest that a genomic variation or a polymorphism in the exposed generation (represented by a, b, and c) plays a role in “losing” or “maintaining” the epigenetic effect in future generations. Partly based on our interpretation of reports discussed in this review, we believe that the interplay between environmentally induced epigenetic changes (also called “primary epimutations” by McCarrey et al. [70]) and (pre-existing) individual genetic characteristics (as we here hypothesize) may induce structural or regulatory changes at the level of the DNA in F2 germ cells, resulting in failure to erase past messages and—at the same time- inducing alterations in the ability to control genomic integrity. We suggest that future studies on transgenerational effects scrutinize potential synergistic mechanisms where both features—a genetic variability and an exposure-induced epigenetic effect—coactively cause a persistent modification in future generations. A triangle represents yet unknown mechanism involved. Because these effects are indirect consequences of an earlier exposure, it can be interpreted as an adaptation to the new environment. This novel scenario could also contribute to the (unexplained) acceleration in evolution and speciation, earlier discussed in the context of dietary exposures [23]

Alternatively, we believe that the opposite could also be true. Namely, that a variation or polymorphism in DNA sequence (e.g., SNPs, STRP, etc.) could lead to a higher or a lower susceptibility of an individual to pass down the environmental trait to the next generation(s). For instance, single-nucleotide polymorphism (SNPs) could be at the origin of newly established (or removal of) CpG sites in gene-regulatory CpG islands and cause differential methylation profiles. The next generation could inherit the parental SNP and also an exposure-induced epigenetic signature at or around this site. Both features together might synergistically lead to genomic instability in the following generations and cause new phenotypes or diseases. Figure 2 illustrates this hypothesis, which is based on findings and theories described in this review and our own interpretations or additions about the interplay of genetics and epigenetics caused by an ancestral external exposure. As also described McCarrey et al., an initial epimutation may be at the origin of genomic instability, inducing an accelerated genetic mutation; hence, causing a so-called tertiary epimutation in the next generations [70]. However, in their theory, it remained unclear why some primary epimutations are only temporal (leading to intergenerational effects) and why in other cases the initial effects may turn into (or can trigger) a tertiary epimutation, causing persistent transgenerational phenotypes. Hence, in our hypothesis, we define a new determinant that could explain why some of the primary epigenetic effects may lead to responses in future generations. Our hypothesis could be tested by comparing lineages of generations of animals (or humans) that are affected versus those that are not transgenerationally affected by an ancestral exposure. Ultimately, epigenetic polymorphisms could be defined as biomarkers to estimate the risk of transgenerational inheritance of an early exposure.

Furthermore, we suggest that each individual carries some “evolutionary determination” that depends on its DNA profile and the encountered exposures during life, or at specific time-points in life [13]. Variation in findings between different reports may be due to different ways of the exposure, but also to the use of different animal strains. For instance, some SNPs—as well as deletions and insertions—are unique to one specific strain, called “private SNPs” or “private deletions/insertions,” respectively [103]. This should be further investigated in the future.

It should also be noted that a large number of studies were produced by the same research group, led by Skinner. Most of their studies involved VCZ exposures. It is important that their findings are reproduced by others, for instance through different methodological approaches or study designs involving several doses and various windows of exposure. However, Skinner’s combined studies and observations are of important value. They enable to integrate different epigenetic factors, such as DNA methylation, ncRNAs, and histone retentions and modifications. This approach enhances our understanding of the transgenerational inheritance phenomenon. In the current set of selected reports, data on use of various dosages were often limited; hence, epigenetic inherited effects could not always be verified by dose of exposure. Still, it would be difficult to translate dosages from animal experiments to human situations. Humans are exposed to mixtures of chemicals, causing interference that are not comparable to laboratory circumstances; although interesting attempts have been performed in mice to mimic the human situation [76]. Notably, it has been documented that even at very low doses, epigenetic effects can be measured without presence of phenotypic effects [104]. Hence, when it comes to epigenetic (transgenerational) endpoints, the safe lower limit has not been determined yet. It is also important to note heterogeneity of the methodologies used in different studies. Discrepancies in findings can be due to the use of different species or strains (as explained above), the way of administration (orally or ip), window of exposure and/or doses used, and chronicity of the exposure (daily versus single dose). Furthermore, reports differentiate in their transparency regarding protocols and numbers of animals or samples tested, varying between nice representations of breeding schemes to vague descriptions of the methods. Possibly, restrictions in word count or numbers of figures may be at the origin of this issue, but we highly recommend scientific journals to demand their authors providing detailed and clear protocols of their study designs. Only then, quality assessments can be carried out properly and studies can be reproduced.

#### Public health implications and precautions

Our review findings open new perspectives to better estimate exposure risk of EDCs before they are introduced on the market. For instance, including validation of epigenetic inheritance of newly produced EDCs before they reach the market—in whichever form—would prevent unforeseen harmful consequences to human health years after market introduction. In the future, practices of risk assessment and related damage will need a larger scope; inclusion of multi-generation epigenetic tests are needed. However, it is still challenging to guide the industry and policy makers. We urgently need to improve our understanding of EDC-related effects in time (e.g., time window of the exposure, inheritance through generations) and in quantity (dose-response effects); not only from animal models but also from human study designs. Large-scale human studies are needed to translate and confirm findings from animal models. We further expect that this field of research will generate a shift in understanding of the concept of “*responsibility*” in providing information at federal and individual level, to prevent diseases. Next, an overall better understanding of epigenetic inheritance will increase our abilities to help patients in the diagnosis of complex disorders in the clinic. It has become apparent that our environment and life style not only influence our health but also that of our children and (great)grandchildren. Therefore, new precautions and guidelines will need to be taken into account. Consequently, not only future mothers but also young men who are planning to have a child should be informed. Although prevention should be one of the first steps to implement, pollutants are already present and will persist for decades in our environment. Hence, dietary supplements may correct or reverse the (epigenetic) damage that has already occurred, or that will emerge in the next generations. Currently, the pharmaceutical industry is already speculating on selling dietary supplements to improve (sperm) health. While some evidence exists in animal models [105–107], clinical efficacy of the use of supplements in men—and especially on the improvement of his offspring health—has not been proven yet. This is another important reason why human studies are urgently needed.

Notably, EDCs have a broad level of impact. For instance, gut-microbiota are also subject to paternal programming. While this was not investigated in the selected reports, there is some promising research in biomedicine regarding their role in disease development. Preconceptional unhealthy diet of fathers has been shown to alter endocrine and metabolic functions, as well as gut microbiota in offspring rats [108, 109]. The gut microbiome is a key component of microbial metabolism of environmental toxins and thus regulates chemical toxicity. For instance, EDCs from pesticides or food additives may induce hyperglycemia and diabetes through the composition of the gut microbiome [110]. It has been shown that after parental exposure to BPA and ethinyl estradiol the gut flora of the F1 generation of rodents is disturbed, making them susceptible to develop metabolic disorders, inflammatory bowel diseases, and colorectal cancer [111]. Despite these early data, the role of the epigenome in EDC-induced effects on offspring gut microbiota and the risk for disorders in the next generations remains largely unknown.

#### Human studies on epigenetic inheritance of diseases: the possible of the impossible

The lack of human evidence in this review highlights the importance of research in the field of epigenetic epidemiology. Few human studies have been performed in this area, but implementation of inter- and transgenerational studies in humans are not straightforward. However, retrospective studies or large existing cohorts could help researchers understand the mechanisms of action of EDCs on the germ line epigenome and the risk for diseases in human offspring. For instance, longitudinal observational studies, such as the “Avon Longitudinal Study of Parents and Children” (ALSPAC) showed that adolescent sons of fathers who started smoking before puberty are at high risk of being obese [20]. Although the authors did not explore the potentially underlying biological mechanisms, this fascinating finding suggests that cigarette smoke toxins may induce epigenetic changes during prepubertal production of spermatogonia in the testes and affect the next generation [112]. Historical data from the isolated municipality of Överkalix in Sweden have revealed effects of early influences from grandparents to grandchildren. Longevity of grandsons was determined by the paternal grandfather’s diet during pre-puberty [19]. These data suggest that information acquired from the environment in early life, when paternal sperm cells are developing from PGCs to spermatogonia, can be stored and transmitted to the next generations.

The first human evidence for a paternally induced epigenetic effect in offspring originates from our study on the Newborn Epigenetics Study (NEST) cohort. We explored this birth cohort for potential associations between epigenetic changes in the offspring and paternal periconceptional life style or obesity status. Significant differences in DNA methylation were found at DMRs of several imprinted genes, if the father was obese [16, 17]. The NEST data suggested that paternal diet (or lack of exercise) had a harmful effect on the progeny through sperm. In two separate studies, we further found that high body mass index (BMI) or exposure to organophosphates was indeed associated with aberrantly methylated DMRs of imprinted genes in sperm [14, 15]. Furthermore, if men were exposed to a mix of chemicals, the risk for producing a sperm sample that was aberrantly methylated at imprinted genes was increased. This suggests that a cocktail of chemicals from our environment—which is realistic in daily life—induces a higher risk of aberrant epigenetic patterns in the male germ line. This is in accordance with findings in the experiments of Skinner’s group, where rodents were exposed to a mix of EDCs [76]. Other data in humans showed that occupational exposure to BPA causes global sperm DNA methylation aberrancies [113]. However, long-term consequences have not been reported.

Still, population studies have their limitations. Studying three to four generations of humans prospectively is practically unfeasible. For ethical reasons, animal experiments are needed to better understand dose-response mechanisms of inter- or transgenerational epigenetic inheritance, for instance. But, this does not mean that human studies are less important. The current review provides interesting results from animal studies about “distinct epigenetic pathways” explaining and distinguishing inter- from transgenerational inheritance. Although the exact mechanisms have not been revealed yet, most likely genetic instability in the unexposed generations follows epigenetic changes that occurred in the earlier exposed generation. We think the time is ripe to explore these findings in a human setting, even if it involves only the primary/intergenerational epigenetic inheritance. We further conclude that a better understanding of the human epigenetic mechanism of inheritance will become highly relevant to public health and clinical applications.

## Supplementary information

**Additional file 1: Supplementary Table 1.** List of studied endocrine disrupting compounds. Potential EDCs of category 1. These 194 chemicals are also listed as the European Union's priority list for future evaluation of their role in endocrine disruption [33, 34].

## Abbreviations

ALSPAC: Avon Longitudinal Study of Parents and Children
ATZ: Atrazine
B[a]P: Benzo[a]pyrene
BPA: Bisphenol A
DBP: Dibutyl phthalate
DDT: Dichlorodiphenyltrichloroethane
DEHP: Bis(2-ethylhexyl)phthalate
DMRs: DNA methylation regions
DNMTs: DNA methyltransferases
E: Embryonic day
EDCs: Endocrine-disrupting compounds
Ehmt1: Euchromatic histone methyltransferase
HCH: 1,2,3,4,5,6-Hexachlorocyclohexane
ip: Intraperitoneal
lncRNA: Long non-coding RNA
MeDIP: Methylated DNA immunoprecipitation
miRNAs: MicroRNAs
mitosRNAs: Mitochondrial genome-encoded small RNAs
MTBE: Methyl tert-butylether
MXC: Methoxychlor
ncRNAs: Non-coding RNAs
NEST: Newborn Epigenetics Study
OECD: Organization for Economic Cooperation and Development
p,p′-DDE: 1,1-Dichloro-2,2-bis(4-chlorophenyl)ethene
P: Postnatal day
PAHs: Polycyclic aromatic hydrocarbons
PBB: Polybrominated biphenyls
PBC: 2,3′,4,4′,5′-Pentachlorobiphenyl
PBDE: Polybrominated diphenyl ethers
PCB: 2,3′,4,4′,5-Pentachlorobiphenyl
PGCs: Primordial germ cells
Pgr: Progesterone receptor
piRNAs: Piwi-interacting RNA
POHaD: Paternal Origins of Health and Disease
RT-qPCR: Quantitative reverse transcription PCR
sncRNA: Small non-coding RNA
SNP: Single-nucleotide polymorphism
STRP: Short tandem repeat polymorphism
TCDD: 2,3,7,8-Tetrachlorodibenzo-p-dioxin
VCZ: Vinclozolin
WHO: World Health Organization
