# Supplementary material for: Transgenerational epigenetic effects from male exposure to endocrine-disrupting compounds: a systematic review on research in mammals
Source: Clin Epigenetics. 2020 May 12;12:65. doi: 10.1186/s13148-020-00845-1 (PMC7218615; doi:10.1186/s13148-020-00845-1)
Supplement: Supplementary file 1 — Additional file 1: Supplementary Table 1. List of studied endocrine disrupting compounds. Potential EDCs of category 1. These 194 chemicals are also listed as the European Union's priority list for future evaluation of their role in endocrine disruption [33, 34]. [file 13148_2020_845_MOESM1_ESM.docx]

**Supplementary Table 1: List of studied endocrine disrupting compounds.**

| 1. Chlordane |
| --- |
| 1. Chlordane (cis and trans) |
| 1. Kepone (Chlordecone) |
| 1. Mirex |
| 1. Toxaphene (Camphechlor) |
| 1. DDT (Clofenotane) |
| 1. p,p’-DDT |
| 1. Tetrachloro DDT |
| 1. Vinclozolin |
| 1. Maneb |
| 1. Metam Natrium |
| 1. Thiram |
| 1. Zineb |
| 1. Gamma-HCH (Lindane) |
| 1. Linuron (Lorox) |
| 1. Atrazine |
| 1. Acetochlor |
| 1. Alachlor |
| 1. Styrene |
| 1. Hexachlorobenzene (HCB) |
| 1. Butylbenzylphthalate (BBP) |
| 1. Di-(2-ethylhexyl) phthalate (DEHP) |
| 1. Di-n-butylphthalate (DBP) |
| 1. 4,4’-isopropylidendiphenol (Bisphenol A or BPA) |
| 1. PCB |
| 1. 2,2’,4,4’,5,5’-hexachlorobiphenyl (PCB 153) |
| 1. PCB 169 |
| 1. PCB 47 |
| 1. PCB 77 |
| 1. PCB Arochlor 1242 |
| 1. PCB Arochlor 1248 |
| 1. PCB Arochlor 1254 |
| 1. PCB Arochlor 1260 (Clophen A60) |
| 1. Brominated biphenyls (PBBs) |
| 1. 1,2,3,7,8 Pentachlorodibenzodioxin (1,2,3,7,8-PCDD) |
| 1. 2,3,7,8-Tetrachlorodibenzo-p-dioxin  (2,3,7,8-TCDD) |
| 1. 1,2,3,7,8-Pentabromodibenzofuran |
| 1. Tributyltin compounds |
| 1. Tributyltin hydride |
| 1. Tributyl Oxide (bis(tributyltin)oxide) |
| 1. Stannane, tributylmecrylate 2. (Stannane, tributylmethacrylate) |
| 1. Methoxyacrylate tributyltin copolymer |
| 1. Phenol, 2-(tributylstannyl)oxy)carbonyl |
| 1. Stannane, benzoyloxytributyl- |
| 1. Stannane, (1,2- phenylenebis (carbonyloxy))bis(tributyl- |
| 1. Stannane, tributyl(naphthalenyloxy)- 2. Tributyltin naphtalate |
| 1. Stannane, tributyl- , mono(naphthenoyloxy)- |
| 1. Stannane, tributyl (1-oxo-9,12 octadecadienyl)oxy)- |
| 1. Stannane, tributyl((1-oxo-9-octadecenyl)oxy)- |
| 1. Stannane, (1R-(1alpha,4abeta,4b alpha,10a alpha))- Tributyl(((1,2,3,4,4a,4b,5,6,10,10a-decahydro-7-isopropyl- 1,4a-dimethyl-1-phenanthryl)carbonyl)oxy) |
| 1. Stannane, tributylfluoro- |
| 1. Stannane, tributyl ((2-methyl-1-oxo-2propenyl)oxy)- |
| 1. Tributyltincarboxylate |
| 1. Tributyltinnaphthalate |
| 1. Tributyltinpolyethoxylate |
| 1. Tri-n-propyltin chloride (TPrT chloride) |
| 1. Triphenyltin |
| 1. Fentin acetate |
| 1. 3,4-Dichloroaniline |
| 1. Resorcinol |
| 1. Amitrol (Aminotriazol) |
| 1. Nitrofen |
| 1. 4-tert-octylphenol |
| 1. Phenol, nonyl- |
| 1. Benzene, 1,3,4-trichloro-5-(1,1-dimethylethyl)-2-methyl (TTBT) |
| 1. 4-Nitrotoluene |
| 1. Carbaryl |
| 1. Cis-Nonachlor |
| 1. Trans-Nonachlor |
| 1. 1,1,1-trichloro-2,2-bis(4-chloro-phenyl)ethane |
| 1. 3-MeO-o,p'-DDE |
| 1. 3-OH-o,p'-DDT |
| 1. 4-MeO-o,p'-DDE |
| 1. 4-MeO-o,p'-DDT |
| 1. 5-MeO-o,p'-DDD |
| 1. 5-MeO-o,p'-DDE |
| 1. 5-MeO-o,p'-DDT |
| 1. 5-OH-o,p'-DDT |
| 1. m,p'-DDD |
| 1. o,p'-DDA-glycinat (N-[(2-chlorophenyl)4-chlorophenyl)acetyl]glycin) |
| 1. o,p'-DDD |
| 1. o,p'-DDE |
| 1. o,p'-DDMU |
| 1. o,p'-DDT |
| 1. p,p'-DDD |
| 1. p,p'-DDMU |
| 1. p,p'-DDE |
| 1. Procymidon |
| 1. Mancozeb |
| 1. Metiram |
| 1. Beta-HCH (isomer til gamma-HCH = Lindane) |
| 1. Hexachlorocyclo-hexane= HCH (gamma-HCH = Lindane) |
| 1. Ioxynil |
| 1. 1,1,1-trichloro-2,2-bis(4-Hydroxyphenyl)ethane (HPTE) |
| 1. 1,3-Dichloro-2,2-bis(4-methoxy-3- methylphenyl)propane |
| 1. Bis-OH-Methoxychlor(1,1,1- trichloro-2,2-bis(4-hydroxyphenyl)ethane (HTPE)) |
| 1. Methoxychlor |
| 1. p,p'-Methoxychlor |
| 1. Fenitrothion |
| 1. Bifenthrin (Talstar) |
| 1. Cyhalothrin, lambda |
| 1. Deltamethrin |
| 1. Resmethrin |
| 1. Fenarimol |
| 1. Picloram |
| 1. Ketoconazol |
| 1. Metribuzin |
| 1. Terbutryn |
| 1. Ethylene Dibromid (1,2-dibromethan or EDB) |
| 1. Trichlorobenzene |
| 1. Pentachlorobenzene |
| 1. Pentachlorophenol (PCP) |
| 1. 4-octylphenol |
| 1. 4-isooctylphenol |
| 1. Nonylphenolethoxylat |
| 1. Intermediate chain chlorinated paraffins |
| 1. Short chain chlorinated paraffins |
| 1. Dicyclohexyl phthalate (DCHP) |
| 1. Diethyl phthalate (DEP) |
| 1. Phenyl-4-hydroxy-phenylmethane |
| 1. 2,2'-bis(2-(2,3-epoxypropoxy)phenyl)propane (2,2-BPPP) (isomer til BADGE) |
| 1. Epichlorohydrin (3-Chloro-1,2-epoxypropane) |
| 1. 2,4-6-Trichlorobiphenyl |
| 1. 3,4',5-Trichlorobiphenyl |
| 1. 3-Hydroxy-2',3',4',5'- tetrachlorobiphenyl |
| 1. 4,4'-Dihydroxy-2,3,5,6-tetrachlorobiphenyl |
| 1. 4,4'-Dihydroxy-3,3',5,5'-tetrachlorobiphenyl |
| 1. 4-Hydroxy-2',3',4',5'-tetrachlorobiphenyl |
| 1. 4-Hydroxy-2',4',6'-trichlorobiphenyl |
| 1. 4-Hydroxy-2,2’, 5'-trichlorobiphenyl |
| 1. 4-Hydroxy-3,3',4',5'-tetrachlorobiphenyl |
| 1. 4-Hydroxy-3,4’, 5-trichlorobiphenyl |
| 1. 4-OH-2,2',4',5,5'-pentachlorobiphenyl |
| 1. Clophen A30 |
| 1. Clophen A50 |
| 1. Mix of 2,3,4,5-Tetrachlorobiphenyl (PCB 61), 2,2’, 4,5,5'-Octachlorobiphenyl (PCB 101) and 2,2',3,3',4,4',5,5'-Octachlorobiphenyl (PCB 194) |
| 1. PCB 104(2,2',4,6,6'-Penta-chlorobiphenyl) |
| 1. PCB 114 (2,3,4,4',5-Penta-chlorobiphenyl) |
| 1. PCB 122 (2,3,3',4,5-Penta-chlorobiphenyl) |
| 1. PCB 126(3,3',4,4',5-Penta-chlorobiphenyl |
| 1. PCB 128(2,2',3,3',4,4'-Hexachloro-biphenyl) |
| 1. PCB 18 (2,2',5-Tri-chlorobiphenyl) |
| 1. PCB 21 (2,3,4-Trichloro-biphenyl) |
| 1. PCB Aroclor 1016 |
| 1. PCB 105 (2,3,3',4,4' -Penta-chlorobiphenyl) |
| 1. PCB 28 (2,4,4'-Tri-chlorobiphenyl) |
| 1. PCB 52 (2,2';5,5'-Tet ra-chlorobiphenyl) |
| 1. PCB 138(2,2',3,4,4',5'- Hexachlorobiphenyl) |
| 1. PCB180(2,2',3,4,4',5 ,5'- Heptachlorobiphenyl) |
| 1. PCB 118(2,3',4,4',5-Penta-chlorobiphenyl) |
| 1. PCT Aroclor 5442 |
| 1. 3,9-Dihydroxy-benz(a)anthracene (3,9-DBA) |
| 1. 5,6-Cyclopento-1,2-benzanthracene (3,5-CPBA) |
| 1. 3-Methylcholanthrene (3-MC) |
| 1. 7,12-Dimethyl-1,2-benz(a)anthracene (DMBA) |
| 1. Benzo[a]pyrene (BAP) |
| 1. 2,3,7,8-TeBDD (tetrabrominated dibenzodioxin |
| 1. 6-Methyl-1,3,8-trichloro-dibenzofuran |
| 1. 2,4-dichlorophenoxy-butyric acid ( 2,4-DB) |
| 1. Mestranol |
| 1. Boric Acid |
| 1. Nonylphenol (4-NP) |
| 1. Omethoate |
| 1. 4-Cyclohexylphenol |
| 1. Ethyl 4-hydroxybenzoate (Ethylparaben) |
| 1. Di-n-pentylphthalate (DPP) |
| 1. Benzophenone-2 ( 2,2’,4,4’ tetra-hydroxybenzophenon) |
| 1. 2,4-Dihydroxybenzophenon (Benzophenone-1) |
| 1. Mono-n-butylphthalate |
| 1. Quinalphos (Chinalphos) |
| 1. 3-Benzylidene camphor (3-BC) |
| 1. Trifluralin |
| 1. Methyl-tert-butylether (MTBE) |
| 1. tert-Butyl-hydroxyanisole (BHA) |
| 1. Phenol, 1,1,3,3-tetramethyl-4-butylphenol (4-tert-octylphenol) |
| 1. 2,6-cis-Diphenylhexamethyl-cyclotetrasiloxane |
| 1. 4-Methylbenzylidenecamphor |
| 1. Mono-2-ethylhexylphthalate (MEHP) |
| 1. Cyclophosphamide |
| 1. 4,4’-Dihydroxy-benzophenone |
| 1. Chlordimeform |
| 1. p-Coumaric acid (PCA) |
| 1. 3,3’Bis(4-hydroxyphenyl) phthalid (Phenolphthaleine) |
| 1. 2,2-Bis(4-hydroxy-phenyl)-n-butan (Bisphenol B) |
| 1. 4-Hydroxybiphenyl (4-Phenylphenol) |
| 1. 4,4’Dihydroxy-biphenyl |
| 1. n-Propyl p-hydroxybenzoate (Propylparaben) |
| 1. n-Butyl p-hydroxybenzoate (Butylparaben) |
| 1. Dibromochloropropane (DBCP) |
| 1. Ethylene Thiourea (ETU) |
| 1. Methyl p-hydroxybenzoate (Methylparaben) |
| 1. p-Hydroxybenzoic acid |
